# Sparse-Representation-Based Direct Minimum *L* ^*p*^-Norm Algorithm for MRI Phase Unwrapping

**DOI:** 10.1155/2014/134058

**Published:** 2014-03-26

**Authors:** Wei He, Ling Xia, Feng Liu

**Affiliations:** ^1^Department of Biomedical Engineering, Zhejiang University, Hangzhou 310027, China; ^2^School of Information Technology and Electrical Engineering, The University of Queensland, Brisbane, QLD 4072, Australia

## Abstract

A sparse-representation-based direct minimum *L* ^*p*^-norm algorithm is proposed for a two-dimensional MRI phase unwrapping. First, the algorithm converts the weighted-*L* ^*p*^-norm-minimization-based phase unwrapping problem into a linear system problem whose system (coefficient) matrix is a large, symmetric one. Then, the coefficient-matrix is represented in the sparse structure. Finally, standard direct solvers are employed to solve this linear system. Several wrapped phase datasets, including simulated and MR data, were used to evaluate this algorithm's performance. The results demonstrated that the proposed algorithm for unwrapping MRI phase data is reliable and robust.

## 1. Introduction

From the MRI complex data, the phase information can be extracted with a restricted interval (−*π*, *π*]. That is, the phase value is wrapped. We call it a wrapped phase or the principal value *ψ*. This relationship between the wrapped phase and its corresponding true phase *θ* can be described by *ψ* = *ω*{*θ*} = *θ* + 2*kπ*, where *k* is an integer and *ω*{·} is the wrapping operator, forcing the value of its argument inside the curly braces into the range (−*π*, *π*] by adding or subtracting an integral multiple of 2*π* radians from its argument. However, what is needed is the true unknown phase *θ*, because this relates to certain properties of interest, such as the velocity of the moving spins, the main *B*_0_ field inhomogeneity, and the magnetic susceptibility variations. Given the wrapped phase, phase unwrapping is applied to restore the true phase, obtaining the unwrapped estimate *φ*. This technique is an important tool in many MRI applications, for example, three-point Dixon water and fat separation [1], MR venography [2, 3], motion tracking in a tagged cardiac MRI [4], and field mapping in EPI [5].

**Figure 1:**
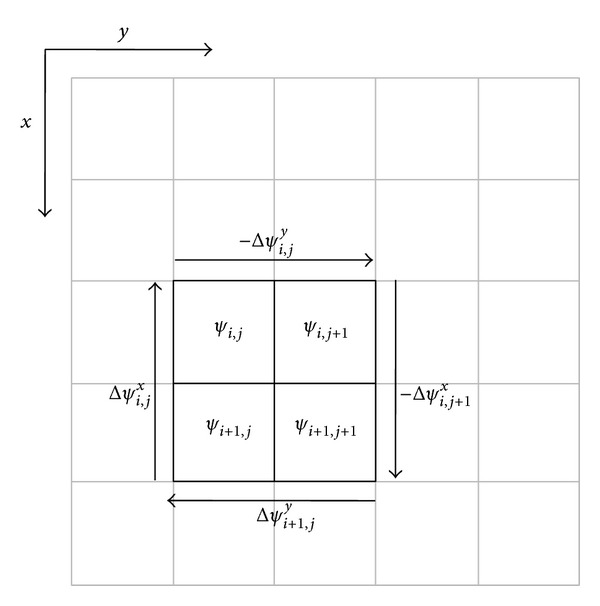
Residue calculation.

Should the wrapped phases have no inconsistencies, the process of the phase unwrapping will be merely integrating the phase gradients over a path that covers the whole domain of interest. This process is quite simple and path independent. No matter which path is followed, the results will be the same regardless of the constant offset. However, in practice, there are always inconsistencies owing to the presence of the noise, undersampling, and/or object discontinuities. Consequently, phase unwrapping becomes intractable and path dependent. The inconsistency is usually called “residue” and is detected by summing the wrapped phase gradients around each 2 × 2 closed loop in the two-dimensional (2D) array, as shown in Figure 1. Consider

(1) $$\begin{array}{c} \Delta \psi_{i,j}^{x}-\Delta \psi_{i,j}^{y}-\Delta \psi_{i,j+1}^{x}+\Delta \psi_{i+1,j}^{y}=\pm 2\pi , \end{array}$$

where Δ*ψ*_*i*,*j*_^*x*^ and Δ*ψ*_*i*,*j*_^*y*^ are defined to be the gradients of the wrapped phases:

(2) $$\begin{array}{c} \Delta \psi_{i,j}^{x}=\omega \left\{\psi_{i+1,j}-\psi_{i,j}\right\},\\ \Delta \psi_{i,j}^{y}=\omega \left\{\psi_{i,j+1}-\psi_{i,j}\right\}. \end{array}$$

In the literature, numerous two-dimensional (2D) phase unwrapping approaches have been developed. They can be classified into four categories: path-following [6–11], minimum-norm [12–18], Bayesian/regularization [19, 20], and parametric modeling [21] methods. Our method belongs to the second category; thus, we briefly introduce the minimum-norm methods.

The minimum-norm methods estimate the unwrapped phases by minimizing the *L* ^*p*^-norm of the differences between the gradients of the wrapped and the unwrapped phases. With *p* = 2, this results in least-square algorithms. These employ the FFT/DCT transforms [18, 22] or/and iterative techniques [22] to reach an approximation for the least-square solution. The exact least-square solution is obtained by applying network programming techniques in [15]. Nevertheless, the least-square minimization tends to smooth the discontinuities, unless these discontinuities are given in advance as binary weights. The minimum *L* ^1^-norm algorithms [13, 23] have a better ability than the *L* ^2^-norm ones to preserve the discontinuities. As *p* approaches zero, the minimum *L* ^*p*^-norm method tends to obtain a more reliable result [24, 25]. Thus, the *L* ^0^-norm minimization is widely accepted as the most desirable in practice. However, the *L* ^0^-norm minimization is a nondeterministic polynomial-time hard (NP-hard) problem [14] with only approximate, not exact, solutions being developed in [12, 14]. The conventional minimum *L* ^*p*^-norm method [12] converts the *L* ^*p*^-norm minimization problem into a generalized matrix equation with a flexible option of *p*. It yields more accurate solutions than other methods, except for the cases including some residues closer to the periphery than to the other residues with opposite polarity. Additionally, because it is implemented in a dual-iterative structure (an iteration structure embedded in an outer one), it can be computationally intense.

In this work, a sparse-representation-based direct minimum *L* ^*p*^-norm (SDM*L* ^*P*^) algorithm is proposed. It introduces the user-defined weights into the generalized matrix equation of the conventional minimum *L* ^*p*^-norm method to improve the performance of phase unwrapping, because, in this way, the discontinuities in the unwrapped phase surface can be confined to the low-quality or zero-weight regions [22]. On the other hand, the sparse representations of the matrices in the modified matrix equation and the direct solvers are exploited in this algorithm to significantly reduce the computational time of the conventional minimum *L* ^*p*^-norm method for MRI phase unwrapping.

## 2. Materials and Methods

### 2.1. Mathematics Foundation

In general, for an *M* × *N* 2D wrapped phase array, the weighted minimum *L* ^*p*^-norm phase unwrapping problem is expressed by [14, 17]

(3) argminφ∑i=1M−1 ∑j=1Nwi,jx·|φi+1,j−φi,j−Δψi,jx|p  +∑i=1M ∑j=1N−1wi,jy·|φi,j+1−φi,j−Δψi,jy|p,

where *w*_*i*,*j*_^*x*^ and *w*_*i*,*j*_^*y*^ are the user-defined weights for corresponding differences and indicate where the phase values are more reliable than others. The user-defined weights are given by

(4) wi,jx=min⁡(wi+1,j,wi,j),i=1,2,…,M−1;  j=1,2,…,N,wi,jy=min⁡(wi,j+1,wi,j),i=1,2,…,M;  j=1,2,…,N−1,

where

(5) $$\begin{array}{c} w_{i,j}=\frac{\max\left(z_{i,j}\right)-z_{i,j}}{\max\left(z_{i,j}\right)-\min\left(z_{i,j}\right)},\\ z_{i,j}=\frac{\sqrt{\sum {\left(\Delta \psi_{i,j}^{x}-\bar{\Delta \psi_{m,n}^{x}}\right)}^{2}}+\sqrt{\sum {\left(\Delta \psi_{i,j}^{y}-\bar{\Delta \psi_{m,n}^{y}}\right)}^{2}}}{l^{2}}. \end{array}$$

For each sum, the indexes (*i*, *j*) cover over the *l* × *l* window centered at the pixel (*m*, *n*). The terms Δ*ψ*_*i*,*j*_^*x*^ and Δ*ψ*_*i*,*j*_^*y*^ are the wrapped phase gradients in the *l* × *l* windows and $\bar{\Delta \psi_{m,n}^{x}}$ and $\bar{\Delta \psi_{m,n}^{y}}$ are the averages of these wrapped phase gradients. In this paper *l* = 3. *w*_*i*,*j*_ is relatively higher in the areas where the phase changes smoothly.

Through the analogous derivation process in [12, 26], the minimum *L* ^*p*^-norm solution of (3) eventually entails the solution of the following linear system of equations:

(6) Ri,j(φi+1,j−φi,j)+Ci,j(φi,j+1−φi,j)  −Ri,j(φi,j−φi−1,j)−Ci,j(φi,j−φi,j−1)=ρi,j,

where

(7) Ri,j=wi,jx·|φi+1,j−φi,j−Δψi,jx|p−2,i=1,2,…,M−1;  j=1,2,…,N,

(8) Ci,j=wi,jy·|φi,j+1−φi,j−Δψi,jy|p−2,i=1,2,…,M;  j=1,2,…,N−1,

(9) $$\begin{array}{c} \rho_{i,j}=R_{i,j}\Delta \psi_{i,j}^{x}-R_{i,j}\Delta \psi_{i-1,j}^{x}+C_{i,j}\Delta \psi_{i,j}^{y}-C_{i,j}\Delta \psi_{i,j-1}^{y}. \end{array}$$

That is to say, when the weighted minimum *L* ^*p*^-norm phase unwrapping solution is desired, it can be simpler to find the solution *φ*_*i*,*j*_ of (6) instead.

For the setting of the values of data-dependent weights *R*_*i*,*j*_ and *C*_*i*,*j*_ empirically, the weights are always normalized in the interval [0, 1] in practice. Hence, (7) and (8) are modified to incorporate this constraint. To avoid |·|^*p*−2^ with 0 ≤ *p* ≤ 2 being extremely great or even infinite, the normalization is defined by

(10) Ri,j=wi,jx·α|φi+1,j−φi,j−Δψi,jx|2−p+α,  i=1,2,…,M−1;  j=1,2,…,N,Ci,j=wi,jy·α|φi,j+1−φi,j−Δψi,jy|2−p+α,  i=1,2,…,M;  j=1,2,…,N−1.

Here, setting *α* to be 0.01 radians is a compromise between accuracy and efficiency [12, 22].

### 2.2. Sparse-Representation-Based Direct Minimum *L* ^*p*^-Norm (SDM*L* ^*P*^) Algorithm

#### 2.2.1. Sparse Representation of the Objective

The estimated phase distribution is shown in Figure 1. To simplify the algorithm, concatenating the columns of the phase matrix yields a vector of length* MN*:

(11) ϕ=[φ1,1,φ2,1,…,φM,1,φ1,2,φ2,2,…,φM,2,…, φ1,N,…,φM,N]T.

The superscript *T* refers to a matrix transpose.

The wrapped phase differences along the two directions, Δ*ψ* ^*x*^ and Δ*ψ* ^*y*^, are also matrices. Their sizes are (*M* − 1) × *N* and *M* × (*N* − 1), respectively. As above, we concatenate the columns of each matrix and then merge these two vectors vertically into a single array:

(12) d=[Δψ1,1x,…,ΔψM−1,1x,…,Δψ1,Nx,…,ΔψM−1,Nx, Δψ1,1y,…,ΔψM,1y,…,Δψ1,N−1y,…,ΔψM,N−1y]T.

The length of *d* is (*M* − 1)*N* + *M*(*N* − 1).

Analogous to the matrix equation used in the weighted least-squares phase unwrapping algorithm [18], the system of equations defined by (6) can be represented in matrix form as follows:

(13) $$\begin{array}{c} Q\phi =s, \end{array}$$

where **Q** denotes an *MN* × *MN* matrix given by

(14) $$\begin{array}{c} Q=A^{T}W^{T}WA, \end{array}$$

(15) $$\begin{array}{c} s=A^{T}W^{T}Wd. \end{array}$$

**A** is a matrix consisting of an (*M* − 1)*N* × *MN* upper partition and an *M*(*N* − 1) × *MN* lower partition:

(16) $$\begin{array}{c} A=\left[\begin{array}{cccccc} D_{1} & 0 & 0 & \cdots & 0 & 0\\ 0 & D_{1} & 0 & \cdots & 0 & 0\\ 0 & 0 & D_{1} & \cdots & 0 & 0\\ \vdots & \vdots & \vdots & \ddots & \vdots & \vdots \\ 0 & 0 & 0 & \cdots & 0 & D_{1}\\ I & -I & 0 & \cdots & 0 & 0\\ 0 & I & -I & \cdots & 0 & 0\\ 0 & 0 & I & \cdots & 0 & 0\\ \vdots & \vdots & \vdots & \ddots & \vdots & \vdots \\ 0 & 0 & 0 & \cdots & I & -I \end{array}\right], \end{array}$$

where **D**_1_ is an (*M* − 1) × *M* matrix given by

(17) $$\begin{array}{c} D_{1}=\left[\begin{array}{cccccc} 1 & -1 & 0 & \cdots & 0 & 0\\ 0 & 1 & -1 & \cdots & 0 & 0\\ 0 & 0 & 1 & \cdots & 0 & 0\\ \vdots & \vdots & \vdots & \ddots & \vdots & \vdots \\ 0 & 0 & 0 & \cdots & 1 & -1 \end{array}\right] \end{array}$$

and **I** is an *M* × *M* identity matrix.

To make the expansion of (13) equal to (6), the matrix **W** ^*T*^ **W** is constructed as

(18) WTW=diag⁡{R1,1,…,RM−1,1,…,R1,N,…,RM−1,N,C1,1,…,CM,1,…C1,N−1,…,CM,N−1},

where diag⁡{·} puts its arguments in order on the main diagonal. It is easy to see that **W** ^*T*^ **W** is an [(*M*−1)×*N*+*M*×(*N*−1)]^2^ matrix.

If these foregoing matrices are stored and operated using a standard matrix structure, this will consume a significant amount of memory and become unfeasible in standard computers. For instance, given a 256 × 256 phase image, the sizes of **A**, **W** ^*T*^ **W**, and **Q** are 130560 × 65536, 130560 × 130560, and 65536 × 65536, respectively. These matrices are obviously too large (exceeding 2^30^) to be stored and manipulated in a program. Fortunately, all these matrices are sparse and can be represented with a sparse structure. We store only nonzero entries of the matrix together with their indexes. That is, a 3-tuple, (*i*, *j*, *a*_*ij*_), is applied to uniquely identify a nonzero entry of the sparse matrix [27, 28], where *i* and *j* are the row and column indexes, respectively, and *a*_*ij*_ denotes the value of the nonzero entry located in (*i*, *j*). Moreover, to make the subsequent computation more efficient, the nonzero entries are ordered first by columns and then by rows. Finally, the size of the original sparse matrix should also be stored.

It should be noted that, henceforth, **A**, **W** ^*T*^ **W**, and **Q** will still refer to the original sparse matrices but will be stored and operated, respectively, in the corresponding sparse structures.

#### 2.2.2. Direct Solver

Equation (14) implies that **Q** is a large sparse, real, symmetric matrix. Thus, (13) becomes a linear system of equations involving the large sparse symmetric coefficient matrix **Q**. To solve this system of equations, considerable effort has been devoted over the past three decades [29]. Among these algorithms, the direct solvers that rely on the explicit factorization of the coefficient matrix are widely used owing to their generality and robustness. Especially for a symmetric coefficient matrix, the direct solvers prefer to adopt the *LDL* ^*T*^ factorization [30], a variant of Gaussian elimination that can also be considered as an alternative form of the Cholesky factorization without extracting the square roots [31]. Under this factorization, the matrix **Q** is decomposed into a lower triangular matrix **L**, a diagonal matrix **D** with 1 × 1 and 2 × 2 blocks, and the conjugate transpose of **L**. Consider

(19) $$\begin{array}{c} Q={LDL}^{T}. \end{array}$$

Then, given the right-hand side *s*, the estimated phases in (13) can be obtained through a forward elimination followed by the backward substitution [28]. The time complexity is O((MN)^1.5^) and the memory complexity is O (MNlog(MN)). For more details of the direct solvers, please see [32].

#### 2.2.3. Implementation of the Algorithm

During the last two decades a number of software packages that implement direct solvers have been developed [33]. If the coefficient matrix is positive definite, CHOLMOD [34] is adopted, because of its rapid computation and relatively small amounts of memory demand. If not, MA57 [35] is strongly recommended.

To accelerate the convergence, the termination condition is set to no residues for the distinctions [12]. First, the distinction is defined by

(20) $$\begin{array}{c} E_{i,j}=\omega \left\{\psi_{i,j}-\varphi_{i,j}\right\},\;i=1,2,\ldots ,M;\;\;j=1,2,\ldots ,N. \end{array}$$

Then, we treat these distinctions similarly to the phase data. Based on the theory that any wrapped phase image can be uniquely unwrapped (with an arbitrary constant offset) if it does not have residues [36], we check whether the *E*_*i*,*j*_ have residues. Given no residues, we unwrap *E*_*i*,*j*_ by a flood-fill algorithm [37, 38] with a centre start point and no branch cuts.

**Step 1**

Choose the start pixel as the point in the location of *i* = round(*M*/2) and *j* = round(*N*/2), where round(·) rounds its argument to the nearest integer. Its phase value is stored as an unwrapped phase value in the solution matrix. The four neighbouring pixels are next unwrapped and their unwrapped phase values are placed in the solution matrix. These four pixels are inserted in the unwrapped list.

**Step 2**

Pick a pixel from the unwrapped list and then eliminate it from the unwrapped list. Unwrap the phase values of its four neighbouring pixels, avoiding pixels that have been unwrapped. Insert these pixels in the unwrapped list and put their unwrapped phase values in the solution matrix.

**Step 3**

Repeat Step 2 until the unwrapped list becomes empty.

Unwrapping *E*_*i*,*j*_ is signified by *ω* ^−1^{*E*_*i*,*j*_}, which is added back to the estimated phase to obtain the final result. Consider

(21) $$\begin{array}{c} \varphi_{i,j}^{\text{final}}=\varphi_{i,j}+\omega^{-1}\left\{E_{i,j}\right\},\;i=1,2,\ldots ,M;\;\;j=1,2,\ldots ,N. \end{array}$$

In summary, the detailed procedure of the SDM*L* ^*P*^ phase unwrapping algorithm is described as below.

**Step 1**

Set the iteration time *k* = 0. Set the value of *p*. Initialize the estimated phases.

**Step 2**

Compute the weights *R*_*i*,*j*_ and *C*_*i*,*j*_ from (10).

**Step 3**

Compute **Q** and *s* from (14) and (15); then solve (13) by the direct solver.

**Step 4**

Compute the distinction *E*_*i*,*j*_ by (20). Check *E*_*i*,*j*_ for residues. If there are no residues, continue. If *k* > *k*_max⁡_, end. Otherwise, set *k* = *k* + 1 and go to Step 2.

**Step 5**

Unwrap *E*_*i*,*j*_ by the flood-fill algorithm, and form the final estimated phases by (21).

#### 2.2.4. Evaluation

The following weighted *L* ^0^ measure is used to evaluate the quality of an unwrapped solution:

(22) ρ=1MN[∑i=1M−1 ∑j=1Nwi,jx|φi+1,j−φi,j−Δψi,jx|0 +∑i=1M ∑j=1N−1wi,jy|φi,j+1−φi,j−Δψi,jy|0].

This counts the ratio of pixels where the gradients of the unwrapped solution mismatch the wrapped phase gradients, which is what we have also minimized with *p* = 0. Therefore, the lower weighted *L* ^0^ measure indicates the better performance.

## 3. Results and Discussion

Several 2D datasets are used to evaluate the performance of the proposed SDM*L* ^*P*^ algorithm. The actual number of the iterations of the SDM*L* ^*P*^ algorithm varies, depending on the loops after which the distinctions of each example become residue free. The limited maximum number of iterations *k*_max⁡_ is set to be 20.

The performance of the proposed algorithm is compared with conventional minimum *L* ^*p*^-norm algorithm [12] and other two widely used 2D algorithms, PUMA [16] and PHUN [10]. Note that we choose *p* = 0 for the first two methods, because *L* ^0^-norm minimization is more well behaved and most desired in practice (explained in Section 1). We merely display the best result we can obtain for the PHUN method in each example, because the behaviour of this algorithm is controlled by many parameters and the optimum values of the parameters vary for each dataset. All these methods are implemented in MATLAB (MathWorks, Natick, MA) on a PC (Intel 2 Quad CPU 2.39 GHz).

### 3.1. Simulated Data

**Figure 2:**
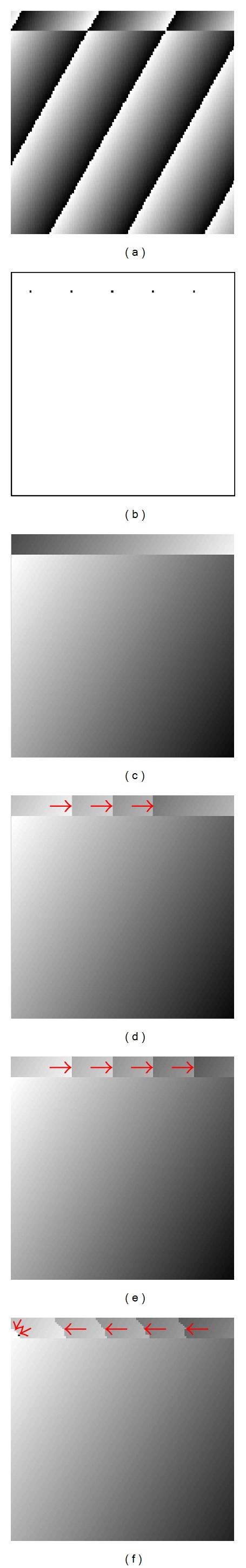
A 128 × 128 phase dataset with shear, (a) its wrapped phase, and (b) its residue distribution including 6 residues. Unwrapped phase image using the (c) SDM*L* ^*P*^, (d) conventional minimum *L* ^*p*^-norm, (e) PUMA, and (f) PHUN methods. Unwrapping errors are marked with red arrows.

We begin with a 128 × 128 phase dataset with one shear line located horizontally along the 13th row below the upper border. The wrapped phase image is shown in Figure 2(a).  Figure 2(b), where the black border is added, shows its residues marked as black dots.

The proposed method, SDM*L* ^*P*^, made no unwrapping errors for this case, producing a solution (see Figure 2(c)) exactly the same as the true phase. However, while it obtained the smallest weighted *L* ^0^ measure for this example, it converged more slowly than the PUMA and PHUN methods (see Figure 6). The conventional minimum *L* ^*p*^-norm and PUMA methods both yielded extra short vertical shear lines marked with red arrows in the upper parts of their results (see Figures 2(d) and 2(e)).  Figure 2(f) shows the solution achieved by the PHUN method with five unexpected crooked shear lines and a black spot of outliers whose values are very low (all marked with red arrows).

**Figure 3:**
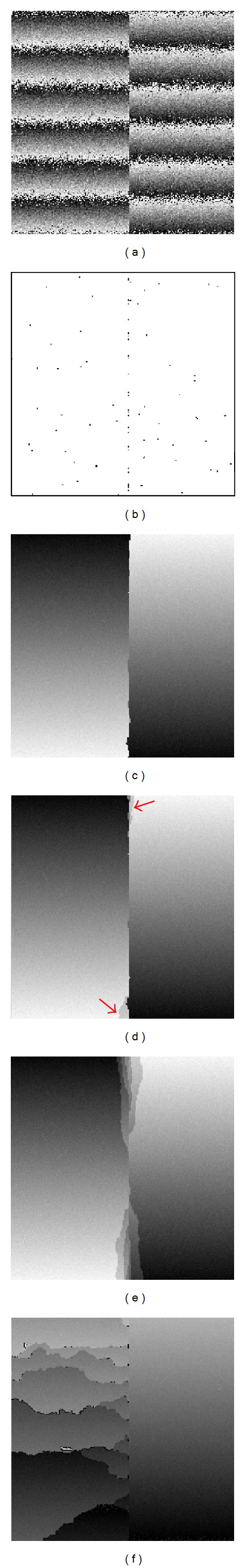
A 257 × 257 noisy phase dataset with shear, (a) its wrapped phase, and (b) its residue distribution including 150 residues. Unwrapped phase image using the (c) SDM*L* ^*P*^, (d) conventional minimum *L* ^*p*^-norm, (e) PUMA, and (f) PHUN methods.

Figure 3(a) shows a 257 × 257 wrapped phase shear image with Gaussian noise (the signal-to-noise ratio is 0.8379 dB). Its residue distribution is shown in Figure 3(b).

The result of the SDM*L* ^*P*^ algorithm in Figure 3(c) is composed of a two-planar surface tearing along the median shear line as expected, except for some protrusions on this line where the phases are severely corrupted by both the noise and the object discontinuities. The conventional minimum *L* ^*p*^-norm method offers a slightly worse result, shown in Figure 3(d), where minor errors (marked with red arrows) occurred near the top and bottom of the median shear line. So the weighted *L* ^0^ measure values of the SDM*L* ^*P*^ and conventional minimum *L* ^*p*^-norm methods are almost the same (see Figure 6(a)). However, it is noted in this case that the former converged much faster than the latter. The unwrapped phase image of the PUMA method in Figure 3(e) has some small-scale, anomalous “layer” artifacts around the median shear line. The PHUN method generated an incorrect unwrapped phase image with the left half full of “layer” artifacts that propagated from the median line to the surrounding regions or even the image border.

In summary, these two simulation examples, both having object discontinuities lying in the shear line, demonstrate the discontinuity-preserving ability of the SDM*L* ^*P*^ algorithm. Additionally, the SDM*L* ^*P*^ algorithm did not produce extra-undesired discontinuities that appeared as shear lines or “layer” artifacts.

### 3.2. MR Data

**Figure 4:**
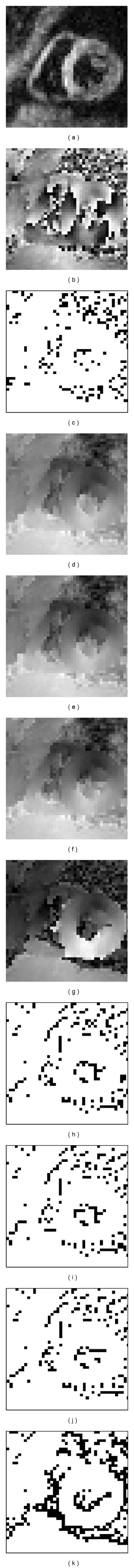
A 44 × 44 displacement encoded MR heart dataset, (a) its magnitudes, (b) its wrapped phases, and (c) its residue distribution including 210 residues. Unwrapped phase image using the (d) SDM*L* ^*P*^, (e) conventional minimum *L* ^*p*^-norm, (f) PUMA, and (g) PHUN methods. Discontinuities in the corresponding unwrapped phase images got by the (h) SDM*L* ^*P*^, (i) conventional minimum *L* ^*p*^-norm, (j) PUMA, and (k) PHUN methods.

Figures 4(a) and 4(b) show the magnitudes and phases of a 44 × 44 displacement encoded MR heart dataset [39], respectively.

**Figure 5:**
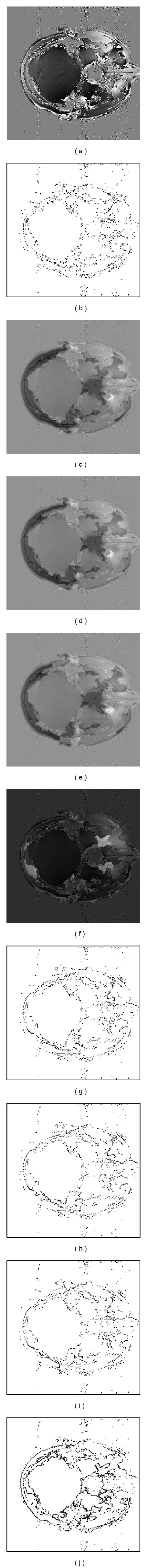
A 256 × 256 MR head dataset, (a) its wrapped phases, and (b) its residue distribution including 1929 residues. Unwrapped phase images using the (c) SDM*L* ^*P*^, (d) conventional minimum *L* ^*p*^-norm, (e) PUMA, and (f) PHUN methods. Discontinuities in the corresponding unwrapped phase images got by the (g) SDM*L* ^*P*^, (h) conventional minimum *L* ^*p*^-norm, (i) PUMA, and (j) PHUN methods.

**Figure 6:**
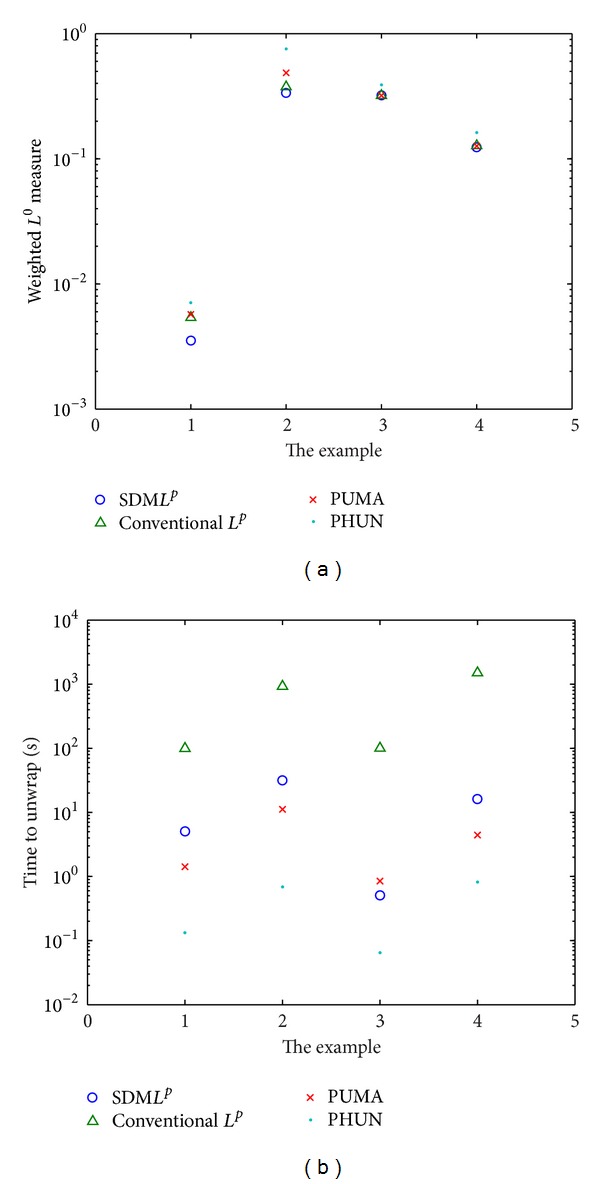
A comparison of the (a) weighted *L* ^0^ measures and (b) execute time of the SDM*L* ^*P*^, conventional minimum *L* ^*p*^-norm, PUMA, and PHUN methods in unwrapping the preceding four datasets.

The results of the SDM*L* ^*p*^, the conventional minimum *L* ^*p*^-norm, and the PUMA algorithms are shown in Figures 4(d), 4(e), and 4(f). There is no significant visible difference between these three images. In these unwrapped phase images, the rough shape of the heart (compared to the magnitude picture in Figure 4(a)) is dimly visible. We then examine the corresponding discontinuity maps (added black borders) that are the distributions of the discontinuities where pixels (marked in black) differ from a neighbouring pixel by more than *π* radians. These maps in Figures 4(h), 4(i), and 4(j) have little differences. In addition, the weighted *L* ^0^ measures, depicted in Figure 6(a), of these three methods are similar. The extremely fast algorithm, PHUN, offered a solution involving many black spots (see Figure 4(g)) that correspond roughly to the locations of the residues (see Figure 4(c)). Also, it created a large number of undesired discontinuities (see Figure 5(j)).

An MR head example [12] is shown in Figure 5. The SDM*L* ^*p*^, the conventional minimum *L* ^*p*^-norm, and the PUMA algorithms all produced plausible unwrapped phase images, shown in Figures 5(c), 5(d), and 5(e). Moreover, the discontinuity maps (see Figures 5(g), 5(h), and 5(i)) and weighted *L* ^0^ measures (see Figure 6(a)) of these three methods are almost the same. The PHUN method failed to unwrap this dataset correctly (see Figure 5(f)). Its result has a large number of undesired discontinuities (see Figure 5(j)).

The weighted *L* ^0^ measures and execute time of all methods in the preceding four examples are compared in Figures 6(a) and 6(b), respectively. For a better visual effect, both of the ordinate axes are in a logarithmic scale. The SDM*L* ^*p*^ algorithm achieved the smallest weighted *L* ^0^ measures for all four examples above. It significantly reduced the computational time compared to the conventional minimum *L* ^*p*^-norm method. However, the algorithm did not converge fast enough compared with the PUMA and PHUN algorithms. From the horizontal comparison of the execute time, it can be concluded that the execute time of the SDM*L* ^*p*^ algorithm depended partly on the size of the phase dataset. For example, the SDM*L* ^*P*^ algorithm converged in 0.5 seconds for the 44 × 44 heart dataset, while, for the 257 × 257 simulated dataset, it took 31 seconds.

**Figure 7:**
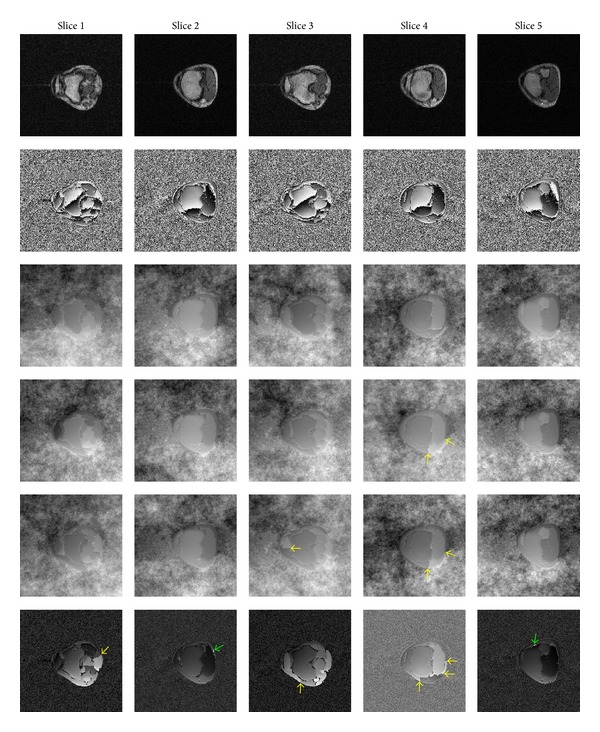
The transverse sections of a 5-slice MR knee dataset. The magnitude and wrapped phase images are shown in the first and second rows, respectively. Unwrapping results of the SDM*L* ^*P*^, conventional minimum *L* ^*p*^-norm, PUMA, and PHUN methods are followed in a top-down order. Undesirable shear lines are marked with yellow arrows. The patches of outliers are marked with green arrows.

The final example is the transverse section of a 5-slice MR knee dataset, as shown in Figure 7. The size of each slice is 256 × 256. The measurements were made with a 0.5T MRI scanner (Ningbo Xingaoyi Co., LTD., China).

Excellent results with no phase errors, shown in the third row of Figure 7, are derived from the unwrapping by the SDM*L* ^*P*^ algorithm. The conventional minimum *L* ^*p*^-norm algorithm worked well, except for generating two undesirable shear lines in the fourth slice. The PUMA algorithm yielded undesired results in the third and fourth slices with some shear lines but successfully unwrapped the other slices. The PHUN method made some unwrapping errors in all slices, shear lines in the first, third, and fourth slices, and white patches of outliers, whose values are very high, in the second and fifth slices. All the truncations and outliers are marked with yellow and green arrows, respectively, in Figure 7.

**Figure 8:**
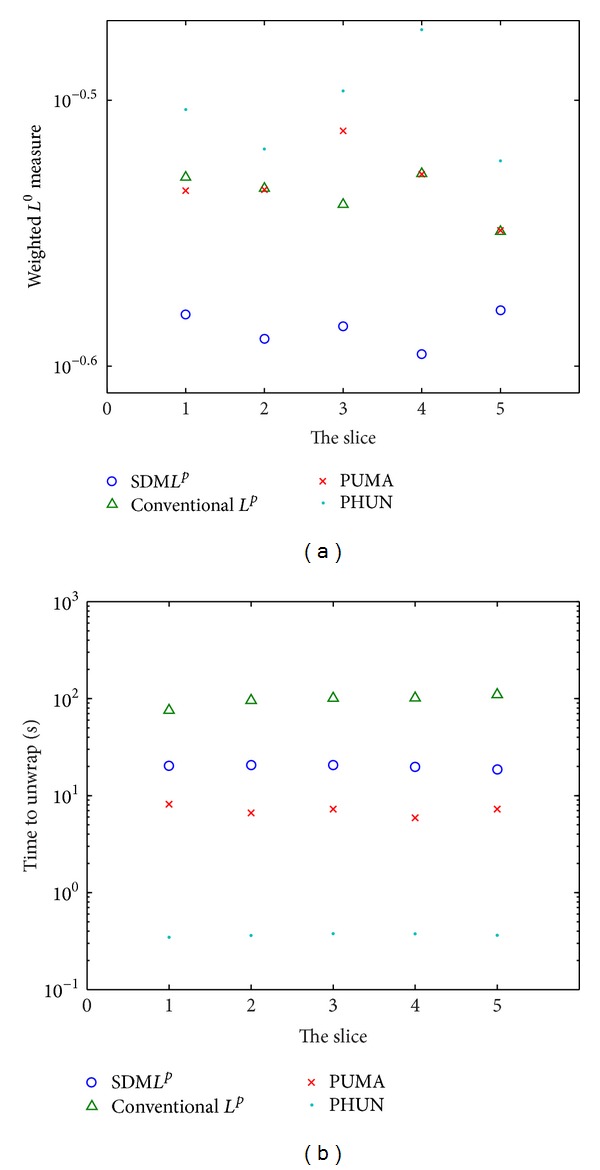
A comparison of the (a) weighted *L* ^0^ measures and (b) execute time of the SDM*L* ^*P*^, conventional minimum *L* ^*p*^-norm, PUMA, and PHUN methods in unwrapping the multislice dataset.

As above, the weighted *L* ^0^ measures and execute time of all the methods for this multislice dataset are compared in Figures 8(a) and 8(b), respectively. The SDM*L* ^*P*^ method returned the smallest weighted *L* ^0^ measures for all slices. Additionally, it yielded these results in 19.62 ± 0.99 seconds, slower than the PUMA and PHUN methods.

## 4. Conclusions

In this work, we developed a sparse-representation-based direct minimum *L* ^*p*^-norm (SDM*L* ^*P*^) algorithm for the 2D phase unwrapping.

The user-defined weights are introduced in the objective function to improve the discontinuity-preserving ability of the SDM*L* ^*P*^ algorithm. Furthermore, the sparse structures are used to represent the matrices involved in the objective function to accelerate the computation and decrease the memory space. Finally, the SDM*L* ^*P*^ algorithms computed effectively and efficiently by employing direct solvers.

The proposed algorithm does produce excellent, reliable results with a very small weighted *L* ^0^ measure; it even allows phase images with large discontinuities through the whole phases to be unwrapped correctly. Moreover, benefiting from using the sparse representation and well-developed direct solvers, the SDM*L* ^*P*^ method converges much faster than the conventional minimum *L* ^*p*^-norm method. However, it is not fast enough. Further research can be devoted to reducing the execution time.

## References

[1] Szumowski J, Coshow WR, Li F, Quinn SF (1994). Phase unwrapping in the three-point Dixon method for fat suppression MR imaging. *Radiology*.

[2] Rauscher A, Barth M, Reichenbach JR, Stollberger R, Moser E (2003). Automated unwrapping of MR phase images applied to BOLD MR-venography at 3 tesla. *Journal of Magnetic Resonance Imaging*.

[3] Bagher-Ebadian H, Jiang Q, Ewing JR (2008). A modified fourier-based phase unwrapping algorithm with an application to MRI venography. *Journal of Magnetic Resonance Imaging*.

[4] Chiang P, Cai Y, Mak KH, Zheng J (2013). A B-spline approach to phase unwrapping in tagged cardiac MRI for motion tracking. *Magnetic Resonance in Medicine*.

[5] Munger P, Greller GR, Peters TM, Pike GB (2000). An inverse problem approach to the correction of distortion in EPI images. *IEEE Transactions on Medical Imaging*.

[6] Su X, Chen W (2004). Reliability-guided phase unwrapping algorithm: a review. *Optics and Lasers in Engineering*.

[7] Herráez MA, Burton DR, Lalor MJ, Gdeisat MA (2002). Fast two-dimensional phase-unwrapping algorithm based on sorting by reliability following a noncontinuous path. *Applied Optics*.

[8] Fang S, Meng L, Wang L, Yang P, Komori M (2011). Quality-guided phase unwrapping algorithm based on reliability evaluation. *Applied Optics*.

[9] Zhou K, Zaitsev M, Bao S (2009). Reliable two-dimensional phase unwrapping method using region growing and local linear estimation. *Magnetic Resonance in Medicine*.

[10] Witoszynskyj S, Rauscher A, Reichenbach JR, Barth M (2009). Phase unwrapping of MR images using ΦUN—a fast and robust region growing algorithm. *Medical Image Analysis*.

[11] Goldstein RM, Zebker HA, Werner CL (1988). Satellite radar interferometry: two-dimensional phase unwrapping. *Radio Science*.

[12] Ghiglia DC, Romero LA (1996). Minimum Lp-norm two-dimensional phase unwrapping. *Journal of the Optical Society of America A*.

[13] Mario Costantini T (1998). A novel phase unwrapping method based on network programming. *IEEE Transactions on Geoscience and Remote Sensing*.

[14] Chen CW, Zebker HA (2000). Network approaches to two-dimensional phase unwrapping: Intractability and two new algorithms. *Journal of the Optical Society of America A*.

[15] Dias JMB, Leitäo JMN (2002). The *ℤπ*M algorithm: a method for interferometric image reconstruction in SAR/SAS. *IEEE Transactions on Image Processing*.

[16] Bioucas-Dias JM, Valadão G (2007). Phase unwrapping via graph cuts. *IEEE Transactions on Image Processing*.

[17] Zhang K, Ge L, Hu Z, Ng AH-M, Li X, Rizos C (2011). Phase unwrapping for very large interferometric data sets. *IEEE Transactions on Geoscience and Remote Sensing*.

[18] Ghiglia DC, Romero LA (1994). Robust two-dimensional weighted and unweighted phase unwrapping that uses fast transforms and iterative methods. *Journal of the Optical Society of America A*.

[19] Nico G, Palubinskas G, Datcu M (2000). Bayesian approaches to phase unwrapping: theoretical study. *IEEE Transactions on Signal Processing*.

[20] Ying L, Liang Z-P, Munson DC, Koetter R, Frey BJ (2006). Unwrapping of MR phase images using a Markov random field model. *IEEE Transactions on Medical Imaging*.

[21] Liang Z-P (1996). A model-based method for phase unwrapping. *IEEE Transactions on Medical Imaging*.

[22] Ghiglia DC, Pritt MD (1998). *Two-Dimensional Phase Unwrapping: Theory, Algorithms, and Software*.

[23] Flynn TJ (1997). Two-dimensional phase unwrapping with minimum weighted discontinuity. *Journal of the Optical Society of America A*.

[24] Dardyk G, Yavneh I (2004). A multigrid approach to two-dimensional phase unwrapping. *Numerical Linear Algebra with Applications*.

[25] Shalem I, Yavneh I (2008). A multilevel graph algorithm for two dimensional phase unwrapping. *Computing and Visualization in Science*.

[26] Mistry P, Braganza S, Kaeli D, Leeser M Accelerating phase unwrapping and affine transformations for optical quadrature microscopy using CUDA.

[27] Tewarson RP (1973). *Sparse Matrices*.

[28] Pooch UW, Nieder A (1973). A survey of indexing techniques for sparse matrices. *ACM Computing Surveys*.

[29] Scott JA, Hu Y (2007). Experiences of sparse direct symmetric solvers. *ACM Transactions on Mathematical Software*.

[30] Watkins DS (2002). *Fundamentals of Matrix Computations*.

[31] Krishnamoorthy A, Menon D Matrix inversion using Cholesky decomposition. http://arxiv.org/abs/1111.4144.

[32] Duff IS, Erisman AM, Reid JK (1986). *Direct Methods for Sparse Matrices*.

[33] Gould NIM, Scott JA, Hu Y (2007). A numerical evaluation of sparse direct solvers for the solution of large sparse symmetric linear systems of equations. *ACM Transactions on Mathematical Software*.

[34] Chen Y, Davis TA, Hager WW, Rajamanickam S (2008). Algorithm 887: CHOLMOD, supernodal sparse cholesky factorization and update/downdate. *ACM Transactions on Mathematical Software*.

[35] Duff IS (2004). MA57—a code for the solution of sparse symmetric definite and indefinite systems. *ACM Transactions on Mathematical Software*.

[36] Chavez S, Xiang Q-S, An L (2002). Understanding phase maps in MRI: a new cutline phase unwrapping method. *IEEE Transactions on Medical Imaging*.

[37] Chen K, Xi J, Yu Y, Chicharo JF Fast quality-guided flood-fill phase unwrapping algorithm for three-dimensional fringe pattern profilometry.

[38] He W, Cheng Y, Xia L, Liu F (2012). A new particle swarm optimization-based method for phase unwrapping of MRI data. *Computational and Mathematical Methods in Medicine*.

[39] Spottiswoode B 2D phase unwrapping algorithms. http://www.mathworks.com/matlabcentral/fileexchange/22504.

